# TeSS: a platform for discovering life-science training opportunities

**DOI:** 10.1093/bioinformatics/btaa047

**Published:** 2020-02-11

**Authors:** Niall Beard, Finn Bacall, Aleksandra Nenadic, Milo Thurston, Carole A Goble, Susanna-Assunta Sansone, Teresa K Attwood

**Affiliations:** b1 Department of Computer Science, The University of Manchester, Manchester M13 9PL, UK; b2 Department of Engineering Science, Oxford e-Research Centre, University of Oxford, Oxford OX1 3QG, UK

## Abstract

**Summary:**

Dispersed across the Internet is an abundance of disparate, disconnected training information, making it hard for researchers to find training opportunities that are relevant to them. To address this issue, we have developed a new platform—TeSS—which aggregates geographically distributed information and presents it in a central, feature-rich portal. Data are gathered automatically from content providers via bespoke scripts. These resources are cross-linked with related data and tools registries, and made available via a search interface, a data API and through widgets.

**Availability and implementation:**

https://tess.elixir-europe.org.

## 1 Introduction

Recent years have witnessed the evolution of a concerted initiative to coordinate the collation and curation of Europe’s life-science data, aiming to provide seamless, open and sustainable access to pivotal datasets that will underpin discoveries of the future—this is ELIXIR (Crosswell and Thornton, 2012). One of ELIXIR’s strategic objectives is to address the known skills gap in bioinformatics and computational biology by providing a comprehensive training programme for professionals. Contributing to the overall training strategy, ELIXIR’s UK ‘Node’ piloted a Training e-Support System (TeSS) for aggregating and discovering training information across Europe and beyond, providing the latest news of ELIXIR-wide training events and resources (Larcombe *et al.*, 2017).

To avoid duplication of effort, TeSS has been designed to provide complementary functionality to, and to work synergistically with, existing training platforms, such as the portal and materials repository developed by the Global Organisation for Bioinformatics Learning, Education and Training (GOBLET) (Attwood *et al.*, 2015; Corpas *et al.*, 2015), the Carpentries (Williams* et al*., 2018) and the Educational Resource Discovery Index (ERuDIte) (Van Horn *et al.*, 2018).

**Fig. 1.**
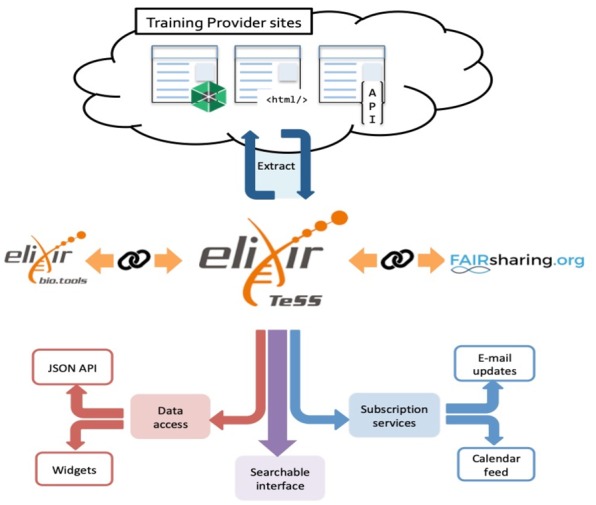
System architecture of TeSS: content is extracted from provider sites, linked with other registries and accessed through various media

Here, we report the current status of TeSS (an overview is shown in Fig. 1), and outline its development plans.

## 2 TeSS features

### 2.1 Search interface and widgets

TeSS provides an easy-to-use interface, including faceted filtering and search features to help users browse and discover training information. TeSS contents are categorized either as training ‘materials’ or training ‘events’: materials are typically slides or recordings from workshops, online tutorials, self-study e-learning modules, etc.; events are generally short-form, time-limited face-to-face or online workshops, e-learning courses, webinars and so on (not full-time academic courses). TeSS collects various metadata properties that can be used for filtering, such as the scientific topic [as defined by the EDAM ontology (Ison *et al.*, 2013)], audience type, difficulty, the tool or database covered, location information and so on.

Communities are often built around specific technologies or professions, or reside in particular geographic areas. Many of these need to disseminate information about relevant training opportunities and events. Rather than directing them to TeSS, we have developed a suite of widgets that can be embedded in any website and configured to display only the information relevant to that particular target community. Adopters of such widgets currently include ISB (www.biocuration.org), GOBLET (www.mygoblet.org) and ABACBS (www.abacbs.org).

### 2.2 Email subscription and calendar feeds

To obviate the need to continuously monitor TeSS for new announcements, users can opt to receive email notifications of new events that match their interests. Alternatively, they can import an overlay onto their calendar application that displays relevant events as listed on TeSS.

### 2.3 Registry integration

Researchers looking to integrate new standards into their work, or to make use of particular tools, can find information about them in other ELIXIR registries, such as bio.tools for tools and web services (https://bio.tools) and FAIRsharing.org for databases, standards and policies (https://fairsharing.org). Often, such registries do not track training relating to the resources they list; TeSS therefore provides the relevant links, allowing users to search for resource-specific training; and the resource pages in bio.tools and FAIRsharing contain reciprocal links for users to discover relevant training in TeSS.

### 2.4 Content acquisition

TeSS allows users to register training events and materials manually. However, this requires significant effort to ensure that information is registered in a timely manner and updated regularly. Consequently, TeSS’ primary mechanism for acquiring training metadata is to aggregate from content providers automatically. Bespoke scrapers are run daily to extract information from a range of target websites: the main techniques involve HTML scraping, application programming interface (API) interfacing and parsing standard structured-data formats (e.g. ICS, RSS, Schema.org): HTML scraping is brittle to host website changes; APIs are more robust, but require construction of custom clients to interface with each provider; use of structured-data formats is optimal, as these adhere to standards.

Many websites do not contain structured data. We encourage providers to mark up their content using the Schema.org specifications for describing common data-types on the web. Schema.org is managed by a consortium of search engines (Google, Bing, Yahoo, etc.), and has huge uptake because its use confers significant search-rank boosts to adopters.

Bioschemas is an initiative that aims to tailor Schema.org specifications to the life sciences. The TeSS platform is at the forefront of developments: we have led the development of new training specifications, and strongly advocate uptake of Schema.org standards for annotating training resources, to facilitate their discovery and interoperability.

## 3 Future work and conclusions

A major challenge for automatic data aggregation from multiple sources is the varying coverage of metadata that each provides: some contain EDAM topic annotations, licence and/or location information; many do not. Using filters in TeSS to hone searches only reveals results containing data relevant to the filter; for more effective searches, more comprehensive metadata are generally required. We are therefore developing semi-automatic metadata inferences by scanning the descriptions of training events for the names of tools, location information and ontology terms to create annotation suggestions where matches occur; these can then be quickly and easily evaluated by human curators.

Other future features focus on surfacing TeSS’ training content in ways that better support user decisions and choices: these include learning paths and training workflows. While both are still works in progress, some early prototypes are currently available from the website.

TeSS offers a ‘shop window’ to ELIXIR’s training landscape: as a central registry, it makes training activities and resources across and beyond Europe discoverable. Tighter coupling between ELIXIR’s core databases/tools/standards and their cognate training resources is facilitated via links with bio.tools and FAIRsharing. Overall, the portal has been shaped by the ELIXIR community; as it matures towards a key ELIXIR service, TeSS will continue to evolve in response to community needs.
